# Prognostic Role of Metabolic Exercise Testing in Heart Failure

**DOI:** 10.3390/jcm12134438

**Published:** 2023-06-30

**Authors:** Arianne Clare Agdamag, Erik H. Van Iterson, W. H. Wilson Tang, J. Emanuel Finet

**Affiliations:** 1 Section of Heart Failure and Transplantation Medicine, Robert and Suzanne Tomsich Department of Cardiovascular Medicine, Miller Family Heart, Vascular and Thoracic Institute, Cleveland Clinic, Cleveland, OH 44195, USA; 2 Section of Preventive Cardiology and Rehabilitation, Robert and Suzanne Tomsich Department of Cardiovascular Medicine, Miller Family Heart, Vascular and Thoracic Institute, Cleveland Clinic, Cleveland, OH 44195, USA

**Keywords:** heart failure, diagnosis, prognosis, cardiopulmonary exercise testing, metabolic exercise testing, oxygen consumption, functional capacity

## Abstract

Heart failure is a clinical syndrome with significant heterogeneity in presentation and severity. Serial risk-stratification and prognostication can guide management decisions, particularly in advanced heart failure, when progression toward advanced therapies or end-of-life care is warranted. Each currently utilized prognostic marker carries its own set of challenges in acquisition, reproducibility, accuracy, and significance. Left ventricular ejection fraction is foundational for heart failure syndrome classification after clinical diagnosis and remains the primary parameter for inclusion in most clinical trials; however, it does not consistently correlate with symptoms and functional capacity, which are also independently prognostic in this patient population. Utilizing the left ventricular ejection fraction as the sole basis of prognostication provides an incomplete characterization of this condition and is prone to misguide medical decision-making when used in isolation. In this review article, we survey and exposit the important role of metabolic exercise testing across the heart failure spectrum, as a complementary diagnostic and prognostic modality. Metabolic exercise testing, also known as cardiopulmonary exercise testing, provides a comprehensive evaluation of the multisystem (i.e., neurological, respiratory, circulatory, and musculoskeletal) response to exercise performance. These differential responses can help identify the predominant contributors to exercise intolerance and exercise symptoms. Additionally, the aerobic exercise capacity (i.e., oxygen consumption during exercise) is directly correlated with overall life expectancy and prognosis in many disease states. Specifically in heart failure patients, metabolic exercise testing provides an accurate, objective, and reproducible assessment of the overall circulatory sufficiency and circulatory reserve during physical stress, being able to isolate the concurrent chronotropic and stroke volume responses for a reliable depiction of the circulatory flow rate in real time.

## 1. Overview of Heart Failure

Heart Failure (HF) is a clinical syndrome that is caused by a structural and/or functional cardiac abnormality and corroborated by elevated natriuretic peptide levels and/or objective evidence of pulmonary or systemic congestion; it affects more than 6.7 million adults in the United States alone [1]. The calculated national cost of HF was USD 30.7 billion in 2012 [2]; due to the aging population, HF has become a growing health and financial burden to the United States and other developed countries [3,4].

In 2022, the ACC/AHA/HFSA endorsed a revised HF classification, dividing patients into four main groups: heart failure with reduced left ventricular ejection fraction (LVEF) ≤ 40% (HFrEF); heart failure with mildly reduced LVEF 41–49% (HFmrEF); heart failure with preserved LVEF ≥ 50% (HFpEF); and heart failure with improved LVEF (HFimpEF), meaning a baseline LVEF of <40% and a subsequent 10-point LVEF increase from baseline and also being >40% [5]. The updated consensus statement also differentiated HF into four stages: Stage A (at risk), Stage B (pre-heart failure), Stage C (current or prior symptoms and/or signs of HF), and stage D (advanced heart failure) [5].

## 2. Risk Prediction in Heart Failure

**Figure 1:**
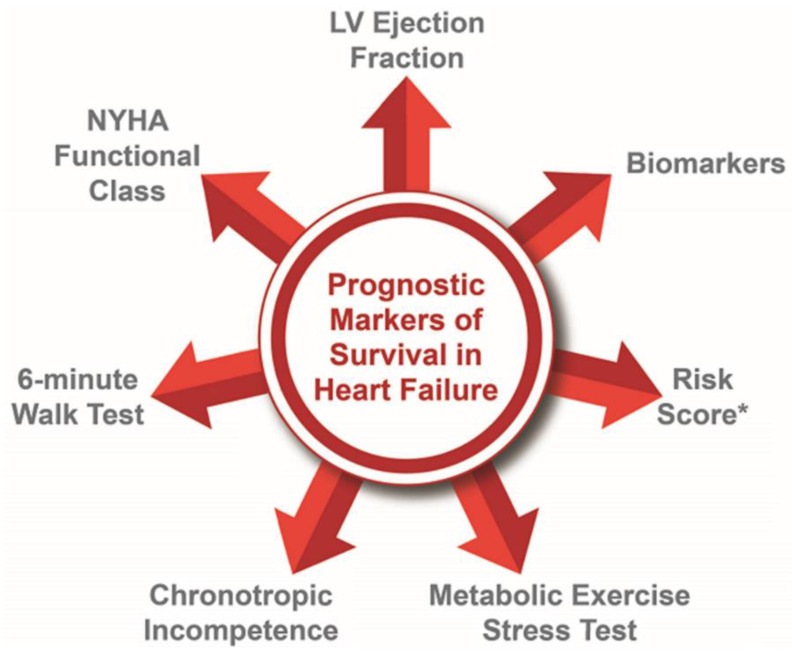
Prognostic Markers for Heart Failure. * Acute Decompensated Heart Failure National Registry-ADHERE [10]; AHA Get With The Guidelines Score [11]; Candesartan in Heart failure-Assessment of Reduction in Mortality and morbidity-CHARM Risk Score [12]; Controlled Rosuvastatin Multinational Trial in Heart Failure-CORONA Risk Score [13]; Enhanced Feedback for Effective Cardiac Treatment-EFFECT Risk Score [14]; Evaluation Study of Congestive Heart Failure and Pulmonary Artery Catheterization Effectiveness-ESCAPE Risk Model and Discharge Score [15]; Guiding Evidence-Based Therapy Using Biomarker Intensified Treatment-GUIDE-IT [16]; Heart Failure Survival Score [17]; Heart Failure: A Controlled Trial Investigating Outcomes of Exercise Training-HF-ACTION [18]; Meta-analysis Global Group in Chronic Heart Failure-MAGGIC [19]; Irbesartan in Heart Failure with Preserved Ejection Fraction-I-PRESERVE Score [20]; PARADIGM-HF [21]; Seattle Heart Failure Model [22]; Treatment of Preserved Cardiac Function Heart Failure with an Aldosterone Antagonist trial-TOPCAT [23].

HF is a clinical syndrome with significant heterogeneity in presentation and severity. Serial risk-stratification and prognostication can guide management decisions, particularly in advanced HF, when progression toward advanced therapies or end-of-life care is warranted [6]. Each of the currently utilized prognostic markers carries its own set of challenges in acquisition, reproducibility, accuracy, and significance; some of them have been further discussed in this work (Figure 1). For example, LVEF is foundational for HF classification after clinical diagnosis and remains the primary parameter for inclusion in most modern HF clinical trials; however, it does not consistently correlate with symptoms or functional capacity [7]. Studies have also shown significant heterogeneity in the 6-minute walk test (6MWT) for the same New York Heart Association (NYHA) class [8,9].

In this review article, we survey and exposit the important role of metabolic exercise testing (MET) across the HF spectrum as a complementary diagnostic and prognostic modality. Also known as cardiopulmonary exercise testing (CPET), MET provides a comprehensive evaluation of the multisystem (i.e., neurological, respiratory, circulatory, and musculoskeletal) response to exercise performance. These differential responses can help identify the predominant contributors to exercise intolerance and exercise symptoms [24]. Additionally, the aerobic exercise capacity (i.e., oxygen consumption during exercise; also known as pVO2) is directly correlated with overall life expectancy and prognosis in many disease states. Specifically in HF patients, MET provides an accurate, objective, and reproducible assessment of the overall circulatory sufficiency and circulatory reserve during physical stress, being able to isolate the concurrent chronotropic and stroke volume responses for a reliable depiction of the circulatory flow rate in real time [25,26].

We subsequently summarize the evidence behind the most common clinical variables currently used as prognostic markers in HF patients.

### 2.1. Left Ventricular Ejection Fraction

Globally utilized as a fundamental clinical-trial inclusion criterion, LVEF is the default variable to initially classify HF syndrome; additionally, its prognostic role has been well-demonstrated [27]. At the present time, LVEF is most commonly measured by echocardiography or cardiac magnetic resonance imaging (MRI), and it is considered decisive in treatment-selection algorithms [28]. However, LVEF does not well represent the underlying pathophysiology of a specific disease process; moreover, heart failure mortality is not directly proportional across the LVEF spectrum [29,30,31,32]. Recent publications have highlighted the need for improved phenotyping among HFrEF patients, given that there is significant heterogeneity in clinical characteristics, outcomes, and responses to therapy [33]. As compared with peak aerobic exercise capacity (pVO2), LVEF has a modest correlation with hemodynamics, functional capacity, and overall prognosis [34,35]. Moreover, LVEF assessment by echocardiography has a high intra- and interobserver variability, with reported values of 8–21% and 6–13%, respectively [36]. Considering these many shortcomings, utilizing LVEF as the sole basis of prognostication provides an incomplete characterization of the HF syndrome, and it is prone to misguide medical decision-making when used in isolation [28,37].

### 2.2. New York Heart Association Functional Class

The New York Heart Association (NYHA) functional classification was first published in 1928 to help physicians communicate patients’ heart-failure symptoms in a shared language. NYHA functional class is widely incorporated in clinical studies, in society guidelines, and in clinical practice; however, patient and physician assessments of symptoms portend to unavoidable subjectivity [7,38,39,40]. It is often difficult to truly assess a patient’s functional capacity and how much heart failure contributes to such symptoms [41,42,43,44,45]. Similarly, Raphael et al. demonstrated that cardiologists have no consistent method of assessing NYHA class and that most research studies do not describe their methods for assigning NYHA class to study participants [43]. Significant heterogeneity of exercise aerobic capacity (pVO2) is also reported within all NYHA classes [7]. Despite these limitations, it is widely used as inclusion or exclusion criteria for therapy, as well as for prognostication and assessment of outcomes [46,47,48].

### 2.3. Six-Minute Walk Test

The 6-minute walk test (6MWT) is an objective, simple, and readily available test to determine functional capacity in heart failure. It is said to be a “poor man’s CPET”. Although it provides an absolute prognostic value, the results are affected by conditions unrelated to the patient’s cardiopulmonary status, such as age, sex, height, and weight, and do not account for physical conditioning [49,50,51]. Despite its inherent limitations, 6MWT does provide a more granular assessment of functional capacity, as compared to the NYHA class, and has been used in predicting outcomes in several conditions, including HF [7,52,53,54].

### 2.4. Chronotropic Incompetence

Chronotropic incompetence is defined as the inability to adequately increase heart rate (HR) commensurate to exercise aerobic capacity. The metabolic chronotropic index (MCI) relates HR reserve to the metabolic reserve at peak exercise (i.e., MCI = [(peak HR − rest HR)/(predicted peak HR − rest HR)]/[(peak VO2 − rest VO2)/(predicted peak VO2 − rest VO2)] [55]. The assessment of MCI can be confounded by commonly used cardiovascular medications, including beta-blockers, ivabradine, and other antiarrhythmic agents [56]; modified criteria have been described in determining MCI in this patient population [57]. Chronic atrial fibrillation and pacemaker dependency can also make the diagnosis of chronotropic incompetence more challenging [58]. Once diagnosed, this abnormal heart rate reserve is associated with reduced functional capacity, worse survival, and increased all-cause hospitalization in patients with HF [59,60,61].

### 2.5. Risk Score Models

Several risk score models have been used to prognosticate patients for chronic HFrEF, acute decompensated HFrEF, and HFpEF and are listed in Figure 1. Risk score models that incorporate metabolic exercise test parameters, including peak VO2, such as MECKI (Metabolic Exercise test data combined with Cardiac and Kidney Indexes) and HFSS (Heart Failure Survival Score), provide the most accurate HF risk prediction and are recommended when assessing patients for transplant listing [62,63]. These risk score models require the collection of multiple variables and may have certain variability in terms of prognostication; however, these risk score models in conjunction with other testing can help guide management decisions [64].

### 2.6. Cardiac Biomarkers

Natriuretic peptides (NPs) are released by cardiac myocytes to maintain circulatory homeostasis. NPs are secreted in response to myocardial tension and increased intravascular volume [65]. N-terminal pro-Brain Natriuretic Peptide (NT-proBNP), which is more sensitive than Brain Natriuretic Peptide, has been consistently associated with increased risk for all-cause mortality and hospitalizations among HF patients regardless of clinical volume status [66]. Biomarkers that alter collagen turnover, cardiac fibrosis, and inflammation may also have diagnostic and predictive value in both HFpEF and HfrEF [67]. Galectin-3, CT-1, GDF-15, and sST2 have been assessed to be the best candidates for determining the early stage of HF development [68,69,70,71,72]. Impaired renal function based on the estimated glomerular filtration rate calculated from the simplified modification of diet in renal disease (MDRD) formula is independently associated with increased risk of all-cause death, cardiovascular death, and hospitalization for HF patients regardless of LVEF [73].

## 3. Overview of Metabolic Exercise Test

Due to the challenges in assessing HF severity with the parameters previously mentioned, there is a need for a more precise definition of hemodynamic involvement and refined prognostic assessment of HF [8]. Metabolic exercise testing (MET) provides a more objective and consistent way of assessing symptom and HF severity and is regarded as the gold-standard for the assessment of functional (aerobic) capacity [25,26,74].

MET allows for an integrated physiological assessment of the pulmonary, cardiovascular, muscular, and cellular oxidative systems [75]. The test allows clinicians to differentiate cardiac from pulmonary disorders, provide outcome prediction, and determine targeted therapies [76,77]. Its easy reproducibility and safe technique make it a suitable choice in the assessment for most patients with undifferentiated symptoms [78,79]. MET also reports variables incorporated in a standard electrocardiogram (ECG) exercise stress test. These include stress and recovery HR and blood pressure (BP), exercise time, exercise workload expressed as metabolic equivalent, patient-reported symptoms, and ECG changes, among others [80]. In conjunction with MET parameters described below, these variables obtained during a standardized exercise stress also confer prognostic significance. Severe findings, such as exercise-induced hypotension, abnormal heart rate recovery, decreased exercise duration, arrhythmias, and angina, all denote a higher risk of cardiac events [81]. Exercise aerobic capacity exhibits the strongest association with all-cause mortality and cardiac events [82].

Current consensus statements and guidelines provide clinical indications for the use of MET, including the assessment of unexplained dyspnea and exercise intolerance, timing of intervention for valvular or congenital heart disease, clinical-trial initiation, and grading severity and prognosticating established advanced cardiac or pulmonary disease [76,83,84].

### 3.1. Performing the Metabolic Exercise Test

The American Thoracic Society/American College of Chest Physicians guidelines on cardiopulmonary exercise testing (i.e., MET) describes full procedural and operational standards [85]. As part of MET preparation among patients with HF, it is common to instruct patients to take all of their standard-of-care medications prior to the test in order to best evaluate the typical medicated integrative physiological response to exercise [86]. Patients typically perform MET on a treadmill or upright cycle ergometer at an increasing workload until maximal exhaustion is achieved or other clinical-test-terminating indications are observed. A patient wears a nose clip and a mouthpiece or full facemask to ensure that continuous breath-to-breath gas exchange and ventilation measurements occur in real time, in a closed-system, for subsequent analysis [87,88]. The use of a cycle ergometer allows for the direct quantification of workload that increases in a ramp-slope or minute-to-minute pattern, whereas the use of a treadmill allows for an indirect estimation of workload according to incremental changes in the treadmill belt slope and speed [89,90]. The test is to be discontinued in the setting of exhaustion, severe arrhythmias, hypotension, angina, and/or severe symptoms, to name a few [81,89]. Documentation of the reason for termination and assessment of dyspnea and perceived effort is to be recorded using subjective scales, such as the modified Borg CR 10 scale [91].

### 3.2. Variables Obtained in Metabolic Exercise Test

The RER refers to the respiratory exchange ratio and is calculated by dividing the carbon dioxide output (VCO2) variable by VO2. An RER ≥ 1.10 is used as one of the main MET features to indicate maximal patient physiological effort during MET [90]. For cardiovascular applications, both maximal and submaximal parameters have been described for clinical purposes. Maximal parameters include peak oxygen consumption (peak VO2), peak circulatory power (CP) (peak VO2 × peak systolic blood pressure), peak VO2 pulse (peak VO2/peak heart rate), and percentage of predicted peak VO2 (%PPVO2), among others [92,93,94]. Submaximal parameters include ventilatory efficiency (i.e., the slope of minute ventilation to CO_2_ production; also know as VE/VCO2 slope), VO2 at the ventilatory-derived anaerobic threshold (VO2@AT), oxygen uptake efficiency slope (slope of the relationship between peak VO2 and log minute ventilation; also known as OUES), end tidal pressure of CO_2_ (PEtCO2), and presence of oscillatory ventilation (EOV), among others [94,95,96,97,98,99,100,101,102,103,104]. Normal expected values for these variables are listed in Table 1, which, alongside the Wasserman–Hansen equations for predicting normal response levels, are generally accepted for use in modern cardiovascular applications [105]. However, because of various patient and data sampling factors that are associated with deriving predicted normal values for MET variables [106], some patient cases may require the referencing of other equation sets, such as those developed from the Fitness Registry and the Importance of Exercise National Database (FRIEND) registry [107].

## 4. Metabolic Exercise Test in Patients with Heart Failure

### 4.1. VO2

Peak VO2 (pVO2) pertains to the maximum consumption of oxygen during exercise. It is the most objective method to assess functional aerobic capacity [83]. Peak VO2 is determined by the interdependent actions of pulmonary respiration, oxygen diffusion at the alveolocapillary membrane, oxygen transport by the cardiovascular system, oxygen diffusion at peripheral capillary beds, and oxygen utilization via the Krebs cycle and the electron transport chain. When there is a reduction in muscle oxygen supply, together with increasing demands for oxygen, this can be a major cause for an inability to achieve a normal pVO2 based on age, gender, and weight [76,108]. Among patients with HF, low O2 delivery can serve as the primary rate-limiting step which may allow for VO2 to be interpreted as a surrogate marker of cardiovascular capacity [109]. Cutoff values for normal pVO2 are dependent on patient age, sex, and body mass index, with the consideration of using lean body mass for obese individuals [110].

Weber et al. introduced the classification of patients with HFrEF based on a pVO2 ranging from A (pVO2 > 20 mL/kg/min) to B (pVO2 15–20 mL/kg/min) to C (pVO2 10–15 mL/kg/min) to D (pVO2 < 10 mL/kg/min) [111]. Mancini et al. subsequently published a landmark study illustrating that, among patients with HFrEF, the established pVO2 “abnormality” cutoff of <14 mL/kg/min was associated with significantly lower 1-year survival as compared to patients who underwent orthotopic heart transplantation (OHT) [34]. Similar observations were subsequently reported among patients on chronic beta-blocker therapy, suggesting a cutoff of <12 mL/kg/min [112,113]. Other groups have consistently reported data supporting the view that pVO2 is a strong univariate predictor of mortality in heart-failure patients [114]. An extremely low pVO2 (i.e., <10 mL/kg/min), when coupled with an abnormal exercise cardiac output response, has been shown to convey the worst prognosis in the HF population [35].

In the HF-ACTION trial, pVO2, percent predicted pVO2, and exercise duration all demonstrated the highest ability to predict mortality in HfrEF [115]. Similar observations have been reported in patients with HfpEF [116,117]. Additionally, in a secondary analysis of the HF-ACTION trial data, a modest increase in pVO2 over 3 months was associated with a lower composite rate of all-cause mortality and hospitalization even with the use of optimal medical and device therapy in HfrEF [118]. The effect of cardiac resynchronization therapy on pVO2 was investigated in the COMPANION trial sub-study, with the results showing no significant improvement in pVO2 at 6 months compared to those on guideline-directed medical therapy [119].

Other measurements which incorporate VO2 can also demonstrate prognostic power in heart-failure patients, even in submaximal aerobic efforts (RER < 1.1). The absolute VO2 response at the ventilatory-derived anaerobic threshold is a useful submaximal exercise prognostic marker; a value of <11 mL/kg/min is associated with higher mortality among HFrEF patients [120]. Flattening of the VO2/HR slope has also been recognized as an indication of inability to augment stroke volume in response to increasing metabolic demand [121]. In repeated MET testing, a >6% pVO2 reduction has been deemed to be high risk [84].

Nadruz et al. published data that pVO2 was independently associated with the total number of HF hospitalizations in all LVEF categories. He also reported that the pVO2 and VE/VCO2 slope provided an independent and incremental prognostic value for all-cause death, LVAD implantation, or heart transplant [41]. These variables also predicted incident HF hospitalization in HFpEF patients. However, when studied in HFmrEF patients, outcomes were only intermediate [41]. Among HFpEF patients, the percentage-predicted pVO2 has been identified as in important prognostic marker [122]. The ESC position paper uses a percentage-predicted pVO2 cutoff of ≥50% as a marker for very low-risk patients [84].

### 4.2. VE/VCO2 Slope

A high VE/VCO2 slope indicates the presence of ventilatory inefficiency, as indicated by a disproportionately high rate of rise in VE relative to that of VCO2, and the coincidence with a sharp fall in arterial CO_2_ associated with hypocapnia [75,108]. The increased VE/VCO2 during exercise in HF is attributed to the combination of hyperventilation and increased dead space ventilation [123]. The increased ventilatory drive can also be attributed to the increased activation of mechano-sensitive j-receptors in response to the abnormal distension of pulmonary vasculature that occurs in pulmonary congestion [124]. A low cardiac index can also be associated with a high VE/VCO2 slope due to the presence of a high V/Q mismatch due to decreased transpulmonary flow [123,125].

An elevated VE/VCO2 slope is associated with an increased pulmonary vascular resistance and inversely associated with a right ventricular ejection fraction in HfrEF [126]. A VE/VCO2 slope > 34, and much more when greater than 45, indicates higher-risk heart-failure patients and predicts worse prognosis in both HFrEF and HFpEF patients [97,127,128,129]. The VE/VCO2 slope has also been found to be independently associated with the total number of HF hospitalizations in the HFrEF population [41]. A comparison with invasive hemodynamics showed how the VE/VCO2 slope obtained during submaximal exercise closely reflects cardiac output, pulmonary artery pressure, and pulmonary capillary wedge pressure [130].

Several studies have suggested a greater value of the VE/VCO2 slope over pVO2 for HF prognosis [97,127,128]. The VE/VCO2 slope provides a better assessment of the complex interplay of pulmonary, cardiac, and peripheral manifestations of heart failure [131]. This parameter is also less likely to fluctuate and is less dependent on the patient’s level of effort as compared to pVO2 [132]. Among the submaximal exercise parameters included in the REVIVAL study analysis, the VE/VCO2 slope is the most reliable and strongest MET predictor of poor outcome within 1 year (durable mechanical circulatory support (MCS) implantation, OHT, or mortality) [133].

### 4.3. Exercise Oscillatory Ventilation

Exercise oscillatory ventilation (EOV) is a form of irregularly irregular cyclic variation of minute ventilation during exercise [134]. EOV can be seen in HF patients regardless of LVEF and indicates a poor prognosis [104,134,135]. EOV is thought to arise from the instability of the feedback systems controlling ventilation similar to Cheyne–Stokes breathing. This can be due to increased circulation time due to a reduced cardiac index, increased chemosensitivity to PaCO2, or baroreflex impairment [136]. EOV can be indicative of autonomic dysregulation and the presence of high chronic levels of sympatho-excitation. Patients with EOV have been found to have clinical characteristics and exercise ventilatory responses consistent with more advanced HF compared to patients with similar LV function [103].

### 4.4. Circulatory Power

Circulatory power (CP) was introduced by Cohen-Solal et al. and is calculated as the product of pVO2 and SBP, thereby estimating total work [93]. This formula utilizes pVO2 as a surrogate for cardiac output and systolic blood pressure as a surrogate for MAP. A circulatory power < 3047 mmHg/mL/min/kg was associated with a lower 1-year survival among patients with HfrEF [93]. In the REVIVAL study cohort, CP was found to be the maximal parameter that was most strongly associated with MCS implantation, OHT, or death at 1 year [133]. Circulatory power also had a greater discriminative capacity to predict the combined endpoint of MCS, OHT, or death based the on receiver-operating-characteristic analysis when compared to O2 pulse and pVO2 [6].

### 4.5. VO2/WR Slope and VO2/HR

The VO2/WR slope (also known as aerobic efficiency) describes the relationship between the oxygen consumption of working muscles to the degree of work generated by them throughout the exercise test [89]. In normal conditions, it increases linearly, as a progressive workload demands progressive oxygen consumption; however, in HF patients, the pattern is a shallow downward shift that is suggestive of VO2 flattening for a given work-rate due to the inability of the heart to match the oxygen delivery required by the workload [75]. As it relates to this variable, VO2/HR is a surrogate stroke volume, also called pVO2 pulse. This surrogate is based on the Fick assumption that the pVO2 is a surrogate of the peak cardiac output, assuming a “normal” mitochondrial function and O2 extraction by the working skeletal muscle [137]. For both VO2/WR and VO2/HR, a shallower slope indicates cardiovascular dysfunction [138].

### 4.6. Ventilatory Power

Ventilatory power is an index derived from combining the VE/VCO2 slope with systolic blood pressure [131]. Forman et al. demonstrated that ventilatory power is a stronger predictor of cardiac events than the pVO2 and VE/VCO2 slope. When analyzed with circulatory power, the prognostic discrimination of ventilatory power is synergistic. A value of <3.5 mmHg was associated with worse prognosis [131].

### 4.7. Hemodynamic Gain Index

The hemodynamic gain index (HGI) is calculated using the formula [(SBPpeak × HRpeak) − (SBPrest × HRrest)]/(SBPrest × HRrest) and was first described by Vainshelboim et al. Initial studies identified the association between a lower HGI and a greater risk of all-cause mortality [139,140]. Further studies have elucidated that a lower HGI was independently associated with all-cause mortality regardless of sex, BMI, and LVEF [141]. HGI provides an integrated measurement of maximal hemodynamic response during exercise. Because it is derived from the relative gain of the rate-pressure product, it reflects cardiovascular function and myocardial oxygen consumption [83,142]. A lower HGI may be suggestive of impaired cardiac functioning, vascular compliance, and vascular performance [141].

### 4.8. Metabolic Exercise Test in Patients with Mechanical Circulatory Support and Cardiac Transplantation

#### 4.8.1. Orthotopic Heart Transplantation

Data from Mancini et al. proposed a pVO2 < 14 mL/kg/min as the cutoff for cardiac transplantation eligibility in patients who are intolerant to beta-blockers, and a threshold of <10 mL/kg/min for those HFrEF patients on chronic betablocker therapy [34]. Current ISHLT recommendations, however, conversely recommend a pVO2 < 12 mL/kg/min cutoff in the presence of chronic betablockers, and a percent-predicted pVO2 < 50% in patients <50 years old and women [143]. Moreover, in obese patients (i.e., BMI > 30 kg/m^2^), adjusting the pVO2 to lean body mass may be considered; a lean-body-mass-adjusted pVO2 < 19 mL/kg/min can serve as a suitable threshold for cardiac transplantation eligibility [143]. Although there is evidence suggesting the VE/VCO2 slope as a stronger prognostic marker than pVO2 in the HFrEF population, current ACC/AHA guidelines recognize pVO2 as the sole marker of impaired aerobic capacity, enough to trigger heart-transplant consideration [76,97]. Conversely, the ISHLT recommends solely the use of the VE/VCO2 slope in the setting of submaximal effort (RER < 1.05) [76,144,145].

MET parameters post-transplantation demonstrate improvement compared to pre-transplant values; however, they may continue to remain abnormal when compared with age-matched healthy individuals, likely due to circulatory deconditioning and impaired cardiac denervation, limiting acute exercise response [146,147]. Several studies have been inconclusive regarding the prognostic value to MET after cardiac transplantation [148,149,150]. Iglesias et al. identified pVO2, oxygen pulse, and percent predicted pVO2 as being independently associated with hospitalizations after cardiac transplantation [151].

#### 4.8.2. Durable Mechanical Circulatory Support

Several MET parameters have been described to predict outcomes after implantation of durable mechanical circulatory support devices (LVADs). Peak VO2 and OUES were associated with 1-, 3-, and 5-year mortality after LVAD implantation. Also, the VE/VCO2 minimum was associated with 3- and 5-year mortality, while the VE/VCO2 slope was also strongly correlated with 5-year mortality [109]. These pre-implantation MET parameters seem to consistently indicate a suitable long-term survival correlation; however, the association with short-term post-implantation outcomes has been shown to be less consistent and weaker [109]. Grinstein et al. additionally reported the association between pre-implantation VE/VCO2 slope and right ventricular failure and mortality following LVAD implantation [152].

The effect of LVAD implantation on VO2 is uncertain, as studies have demonstrated inconsistent results [153,154,155]. Studies by Mirza et al. and Moss et al., among others, have reported that LVAD-supported patients continued to have abnormal pVO2 and impaired exercise capacity, with similar findings even after adjusting for pump speed [156,157]. Similarly, the pVO2 remains significantly impaired when compared with patients who underwent heart transplantation [158,159]; this could be, in part, explained by the unsupported right ventricle (RV), which is also a determinant in circulatory sufficiency. The association between LV function recovery and pVO2 in LVAD patients has also shown mixed results [160,161,162,163]. In this population, pVO2 is an independent predictor of 3- and 5-year mortality, while the VE/VCO2 maximum was an independent predictor of 3-year mortality [109]. Although very few patients undergo LVAD explantation, the incorporation of pVO2 and other MET parameters can be considered in determining the timing of device explantation [164]. Future studies assessing the utility of MET parameters in listing LVAD patients for transplantation should also be considered [156].

#### 4.8.3. Invasive Metabolic Exercise Test

The invasive MET (iMET) incorporates the use of radial and pulmonary catheters for simultaneous hemodynamic monitoring. With the hemodynamic data provided by iMET, it is possible to dissect the oxygen delivery from the oxygen extraction component from the aerobic capacity (i.e., VO2), evaluating in isolation the cardiovascular and mitochondrial functions of the skeletal muscle, sequentially. It is essential for the diagnosis of mitochondrial myopathies and helpful in the evaluation and diagnosis of other conditions of undifferentiated dyspnea, such as exercise-induced pulmonary arterial hypertension (PAH), exercise-induced HFpEF, preload-dependent limitations to cardiac output, chronic thromboembolic disease (CTED), and chronic thromboembolic pulmonary hypertension (CTEPH), among others [165,166,167]. Among LVAD patients, iMET allows us to assess the ability of the right ventricle (RV) to augment pulmonary artery pressure (PAP) and the right ventricular stroke work index. A PAP plateau at exercise indicates the RV’s inability to accommodate increased blood flow, thereby leading to a poor prognosis in these patients [168]. iMET has a role in prognosticating not only LVAD patients but also PAH patients because it can quantify functional limitations to exercise [169]. Issues with invasive MET include its inherent vascular access complications and technical inaccuracies in reliably obtaining pulmonary hemodynamic tracings despite averaging repeated measurements [75].

#### 4.8.4. Metabolic Exercise Test as Clinical Endpoints

**Figure 2:**
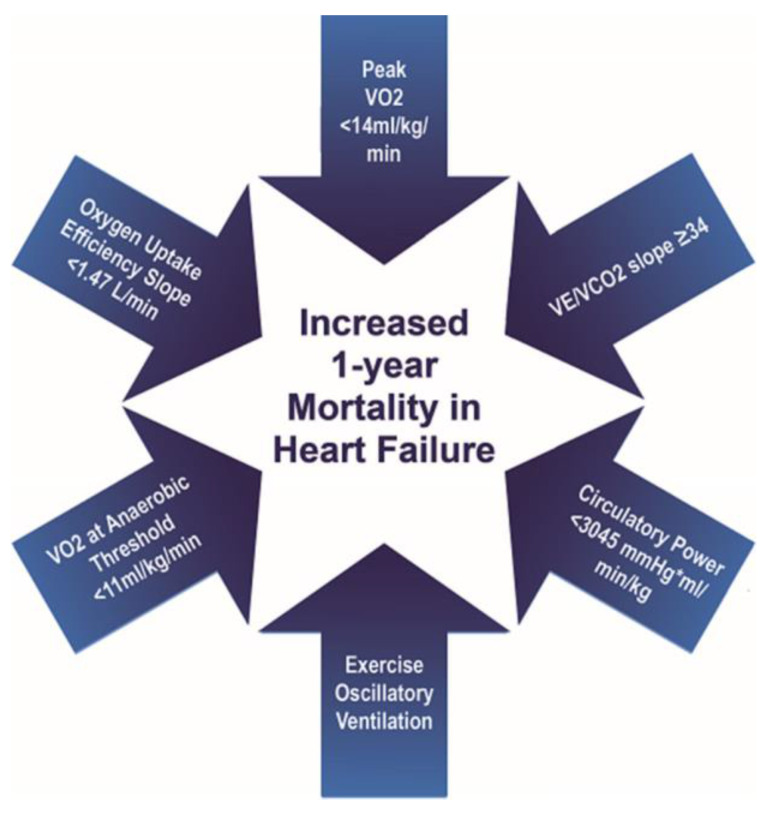
Metabolic Exercise Test Component Threshold.

MET has been used as an endpoint for clinical trials because it allows for the objective measurement of functional capacity at both maximal and submaximal exertion. However, MET incurs a greater overall cost than a submaximal functional or health status assessment and requires expertise in implementation and interpretation. The Heart Failure Collaboratory, and the Academic Research Consortium consensus document indicates that the minimal clinically important difference for maximal VO2 on MET has not been validated. Meaningful difference in peak VO2 has been reported as a 6% change or 1 mL/kg/min but is based on repeat test variability and lack of clinical relevance [170]. Overall, various MET parameters have significant prognostic value in assessing mortality in HF patients regardless of LVEF. Parameters associated with a 1-year mortality risk are summarized in Figure 2.

## 5. Conclusions

Heart failure is a clinical condition with great heterogeneity in presentation, severity, and prognosis. Utilizing LVEF alone to characterize HF will lead to an incomplete assessment of HF severity. MET provides a comprehensive assessment of the integrative multiorgan physiological response to exercise, highlighting its place in the clinician’s evaluation of heart-failure patients. MET data should be incorporated into the characterization of the HF disease state, prognostication, and the decision about the best treatment course for patients.

## Figures and Tables

**Table 1:** MET components and normal values.

Metabolic Exercise Variable	Normal Expected Range
Peak RER	1.10–1.50
Peak work-rate	>85% predicted peak Watts
Peak VO2	>85% predicted peak VO2
Rest VO2	2–5 mL/kg/min
VO2 at VAT	40–75% predicted peak VO2
CI	0.80–1.30
Peak VO2/HR	>85% predicted peak VO2/HR
VO2/WR slope	8.5–12.5 mL/min/watts
Peak SpO2	>95%
Peak VE	<85% predicted peak VE
Peak RR	<60 breaths/min
Peak VT	1.5–3.0 liters
Peak PETCO2	35–41 mmHg
VE/VCO2 slope	<30
EOV	None
Peak RPP	>18,966 mmHg × bpm
HGI	>1.06 bpm/mmHg
Circulatory Power	>3047 mmHg × mL/min/kg
OUES	>1.85 L/min
POUES	>0.88 L/min

RER—respiratory exchange ratio; VO2—peak oxygen consumption; VAT—ventilatory anaerobic threshold; CI—chronotropic index; HR—heart rate; SpO2—oxygen saturation; VE—ventilation, RR—respiratory rate; VT—tidal volume; PETCO2—end-tidal pressure of carbon dioxide; EOV—exercise oscillatory ventilation; RPP—rate pressure product (heart rate × systolic blood pressure); HGI—hemodynamic gain index; OUES—oxygen uptake efficiency slope; POUES—oxygen uptake efficiency at peak exercise.

## Data Availability

No new data was created.

## References

[1] Tsao C.W., Aday A.W., Almarzooq Z.I., Anderson C.A.M., Arora P., Avery C.L., Baker-Smith C.M., Beaton A.Z., Boehme A.K., Buxton A.E. (2023). Heart Disease and Stroke Statistics-2023 Update: A Report from the American Heart Association. Circulation.

[2] Benjamin E.J., Muntner P., Alonso A., Bittencourt M.S., Callaway C.W., Carson A.P., Chamberlain A.M., Chang A.R., Cheng S., Das S.R. (2019). Heart Disease and Stroke Statistics-2019 Update: A Report From the American Heart Association. Circulation.

[3] Heidenreich P.A., Albert N.M., Allen L.A., Bluemke D.A., Butler J., Fonarow G.C., Ikonomidis J.S., Khavjou O., Konstam M.A., Maddox T.M. (2013). Forecasting the impact of heart failure in the United States: A policy statement from the American Heart Association. Circ. Heart Fail..

[4] Virani S.S., Alonso A., Aparicio H.J., Benjamin E.J., Bittencourt M.S., Callaway C.W., Carson A.P., Chamberlain A.M., Cheng S., Delling F.N. (2021). Heart Disease and Stroke Statistics-2021 Update: A Report From the American Heart Association. Circulation.

[5] Bozkurt B., Coats A.J., Tsutsui H., Abdelhamid M., Adamopoulos S., Albert N., Anker S.D., Atherton J., Bohm M., Butler J. (2021). Universal Definition and Classification of Heart Failure: A Report of the Heart Failure Society of America, Heart Failure Association of the European Society of Cardiology, Japanese Heart Failure Society and Writing Committee of the Universal Definition of Heart Failure. J. Card. Fail..

[6] Lewis G.D., Zlotoff D.A. (2021). Cardiopulmonary Exercise Testing-Based Risk Stratification in the Modern Era of Advanced Heart Failure Management. JACC Heart Fail..

[7] Yap J., Lim F.Y., Gao F., Teo L.L., Lam C.S., Yeo K.K. (2015). Correlation of the New York Heart Association Classification and the 6-Minute Walk Distance: A Systematic Review. Clin. Cardiol..

[8] Ambrosio G., Carluccio E. (2018). Prognostic role of left ventricular ejection fraction in heart failure: Back to the future?. Int. J. Cardiol..

[9] Calkins D.R., Rubenstein L.V., Cleary P.D., Davies A.R., Jette A.M., Fink A., Kosecoff J., Young R.T., Brook R.H., Delbanco T.L. (1991). Failure of physicians to recognize functional disability in ambulatory patients. Ann. Intern. Med..

[10] Fonarow G.C., Adams K.F., Abraham W.T., Yancy C.W., Boscardin W.J., Adhere Scientific Advisory Committee, Study Group (2005). Risk stratification for in-hospital mortality in acutely decompensated heart failure: Classification and regression tree analysis. JAMA.

[11] Peterson P.N., Rumsfeld J.S., Liang L., Albert N.M., Hernandez A.F., Peterson E.D., Fonarow G.C., Masoudi F.A. (2010). A validated risk score for in-hospital mortality in patients with heart failure from the American Heart Association get with the guidelines program. Circ. Cardiovasc. Qual. Outcomes.

[12] Pocock S.J., Wang D., Pfeffer M.A., Yusuf S., McMurray J.J., Swedberg K.B., Ostergren J., Michelson E.L., Pieper K.S., Granger C.B. (2006). Predictors of mortality and morbidity in patients with chronic heart failure. Eur. Heart J..

[13] Wedel H., McMurray J.J., Lindberg M., Wikstrand J., Cleland J.G., Cornel J.H., Dunselman P., Hjalmarson A., Kjekshus J., Komajda M. (2009). Predictors of fatal and non-fatal outcomes in the Controlled Rosuvastatin Multinational Trial in Heart Failure (CORONA): Incremental value of apolipoprotein A-1, high-sensitivity C-reactive peptide and N-terminal pro B-type natriuretic peptide. Eur. J. Heart Fail..

[14] Lee D.S., Austin P.C., Rouleau J.L., Liu P.P., Naimark D., Tu J.V. (2003). Predicting mortality among patients hospitalized for heart failure: Derivation and validation of a clinical model. JAMA.

[15] O’Connor C.M., Hasselblad V., Mehta R.H., Tasissa G., Califf R.M., Fiuzat M., Rogers J.G., Leier C.V., Stevenson L.W. (2010). Triage after hospitalization with advanced heart failure: The ESCAPE (Evaluation Study of Congestive Heart Failure and Pulmonary Artery Catheterization Effectiveness) risk model and discharge score. J. Am. Coll. Cardiol..

[16] O’Connor C., Fiuzat M., Mulder H., Coles A., Ahmad T., Ezekowitz J.A., Adams K.F., Pina I.L., Anstrom K.J., Cooper L.S. (2019). Clinical factors related to morbidity and mortality in high-risk heart failure patients: The GUIDE-IT predictive model and risk score. Eur. J. Heart Fail..

[17] Aaronson K.D., Schwartz J.S., Chen T.M., Wong K.L., Goin J.E., Mancini D.M. (1997). Development and prospective validation of a clinical index to predict survival in ambulatory patients referred for cardiac transplant evaluation. Circulation.

[18] O’Connor C.M., Whellan D.J., Wojdyla D., Leifer E., Clare R.M., Ellis S.J., Fine L.J., Fleg J.L., Zannad F., Keteyian S.J. (2012). Factors related to morbidity and mortality in patients with chronic heart failure with systolic dysfunction: The HF-ACTION predictive risk score model. Circ. Heart Fail..

[19] Pocock S.J., Ariti C.A., McMurray J.J., Maggioni A., Kober L., Squire I.B., Swedberg K., Dobson J., Poppe K.K., Whalley G.A. (2013). Predicting survival in heart failure: A risk score based on 39 372 patients from 30 studies. Eur. Heart J..

[20] Komajda M., Carson P.E., Hetzel S., McKelvie R., McMurray J., Ptaszynska A., Zile M.R., Demets D., Massie B.M. (2011). Factors associated with outcome in heart failure with preserved ejection fraction: Findings from the Irbesartan in Heart Failure with Preserved Ejection Fraction Study (I-PRESERVE). Circ. Heart Fail..

[21] Simpson J., Jhund P.S., Lund L.H., Padmanabhan S., Claggett B.L., Shen L., Petrie M.C., Abraham W.T., Desai A.S., Dickstein K. (2020). Prognostic Models Derived in PARADIGM-HF and Validated in ATMOSPHERE and the Swedish Heart Failure Registry to Predict Mortality and Morbidity in Chronic Heart Failure. JAMA Cardiol..

[22] Levy W.C., Mozaffarian D., Linker D.T., Sutradhar S.C., Anker S.D., Cropp A.B., Anand I., Maggioni A., Burton P., Sullivan M.D. (2006). The Seattle Heart Failure Model: Prediction of survival in heart failure. Circulation.

[23] Angraal S., Mortazavi B.J., Gupta A., Khera R., Ahmad T., Desai N.R., Jacoby D.L., Masoudi F.A., Spertus J.A., Krumholz H.M. (2020). Machine Learning Prediction of Mortality and Hospitalization in Heart Failure with Preserved Ejection Fraction. JACC Heart Fail..

[24] Nedeljkovic I., Banovic M., Stepanovic J., Giga V., Djordjevic-Dikic A., Trifunovic D., Nedeljkovic M., Petrovic M., Dobric M., Dikic N. (2016). The combined exercise stress echocardiography and cardiopulmonary exercise test for identification of masked heart failure with preserved ejection fraction in patients with hypertension. Eur. J. Prev. Cardiol..

[25] Weber K.T., Janicki J.S. (1985). Cardiopulmonary exercise testing for evaluation of chronic cardiac failure. Am. J. Cardiol..

[26] Lucas C., Stevenson L.W., Johnson W., Hartley H., Hamilton M.A., Walden J., Lem V., Eagen-Bengsten E. (1999). The 6-min walk and peak oxygen consumption in advanced heart failure: Aerobic capacity and survival. Am. Heart J..

[27] Smart N., Haluska B., Leano R., Case C., Mottram P.M., Marwick T.H. (2005). Determinants of functional capacity in patients with chronic heart failure: Role of filling pressure and systolic and diastolic function. Am. Heart J..

[28] Savarese G., Stolfo D., Sinagra G., Lund L.H. (2022). Heart failure with mid-range or mildly reduced ejection fraction. Nat. Rev. Cardiol..

[29] Lam C.S.P., Yancy C. (2021). Universal Definition and Classification of Heart Failure: Is It universal?. Does It Define Heart Failure? J. Card. Fail..

[30] Wehner G.J., Jing L., Haggerty C.M., Suever J.D., Leader J.B., Hartzel D.N., Kirchner H.L., Manus J.N.A., James N., Ayar Z. (2020). Routinely reported ejection fraction and mortality in clinical practice: Where does the nadir of risk lie?. Eur. Heart J..

[31] Solomon S.D., Anavekar N., Skali H., McMurray J.J., Swedberg K., Yusuf S., Granger C.B., Michelson E.L., Wang D., Pocock S. (2005). Influence of ejection fraction on cardiovascular outcomes in a broad spectrum of heart failure patients. Circulation.

[32] Curtis J.P., Sokol S.I., Wang Y., Rathore S.S., Ko D.T., Jadbabaie F., Portnay E.L., Marshalko S.J., Radford M.J., Krumholz H.M. (2003). The association of left ventricular ejection fraction, mortality, and cause of death in stable outpatients with heart failure. J. Am. Coll. Cardiol..

[33] Ahmad T., Pencina M.J., Schulte P.J., O’Brien E., Whellan D.J., Pina I.L., Kitzman D.W., Lee K.L., O’Connor C.M., Felker G.M. (2014). Clinical implications of chronic heart failure phenotypes defined by cluster analysis. J. Am. Coll. Cardiol..

[34] Mancini D.M., Eisen H., Kussmaul W., Mull R., Edmunds L.H., Wilson J.R. (1991). Value of peak exercise oxygen consumption for optimal timing of cardiac transplantation in ambulatory patients with heart failure. Circulation.

[35] Chomsky D.B., Lang C.C., Rayos G.H., Shyr Y., Yeoh T.K., Pierson R.N., Davis S.F., Wilson J.R. (1996). Hemodynamic exercise testing. A valuable tool in the selection of cardiac transplantation candidates. Circulation.

[36] McGowan J.H., Cleland J.G. (2003). Reliability of reporting left ventricular systolic function by echocardiography: A systematic review of 3 methods. Am. Heart J..

[37] Konstam M.A., Abboud F.M. (2017). Ejection Fraction: Misunderstood and Overrated (Changing the Paradigm in Categorizing Heart Failure). Circulation.

[38] Yancy C.W., Jessup M., Bozkurt B., Butler J., Casey D.E., Colvin M.M., Drazner M.H., Filippatos G.S., Fonarow G.C., Givertz M.M. (2017). 2017 ACC/AHA/HFSA Focused Update of the 2013 ACCF/AHA Guideline for the Management of Heart Failure: A Report of the American College of Cardiology/American Heart Association Task Force on Clinical Practice Guidelines and the Heart Failure Society of America. Circulation.

[39] Ponikowski P., Voors A.A., Anker S.D., Bueno H., Cleland J.G.F., Coats A.J.S., Falk V., Gonzalez-Juanatey J.R., Harjola V.P., Jankowska E.A. (2016). 2016 ESC Guidelines for the diagnosis and treatment of acute and chronic heart failure: The Task Force for the diagnosis and treatment of acute and chronic heart failure of the European Society of Cardiology (ESC)Developed with the special contribution of the Heart Failure Association (HFA) of the ESC. Eur. Heart J..

[40] Dunselman P.H., Kuntze C.E., van Bruggen A., Beekhuis H., Piers B., Scaf A.H., Wesseling H., Lie K.I. (1988). Value of New York Heart Association classification, radionuclide ventriculography, and cardiopulmonary exercise tests for selection of patients for congestive heart failure studies. Am. Heart J..

[41] Nadruz W., West E., Sengelov M., Santos M., Groarke J.D., Forman D.E., Claggett B., Skali H., Shah A.M. (2017). Prognostic Value of Cardiopulmonary Exercise Testing in Heart Failure with Reduced, Midrange, and Preserved Ejection Fraction. J. Am. Heart Assoc..

[42] Guazzi M. (2016). Cardiopulmonary exercise testing in heart failure preserved ejection fraction: Time to expand the paradigm in the prognostic algorithm. Am. Heart J..

[43] Raphael C., Briscoe C., Davies J., Ian Whinnett Z., Manisty C., Sutton R., Mayet J., Francis D.P. (2007). Limitations of the New York Heart Association functional classification system and self-reported walking distances in chronic heart failure. Heart.

[44] Severo M., Gaio R., Lourenco P., Alvelos M., Bettencourt P., Azevedo A. (2011). Indirect calibration between clinical observers-application to the New York Heart Association functional classification system. BMC Res. Notes.

[45] Goode K.M., Nabb S., Cleland J.G., Clark A.L. (2008). A comparison of patient and physician-rated New York Heart Association class in a community-based heart failure clinic. J. Card. Fail..

[46] Castel M.A., Magnani S., Mont L., Roig E., Tamborero D., Mendez-Zurita F., Femenia J.F., Tolosana J.M., Perez-Villa F., Brugada J. (2010). Survival in New York Heart Association class IV heart failure patients treated with cardiac resynchronization therapy compared with patients on optimal pharmacological treatment. Europace.

[47] Muntwyler J., Abetel G., Gruner C., Follath F. (2002). One-year mortality among unselected outpatients with heart failure. Eur. Heart J..

[48] Glikson M., Nielsen J.C., Kronborg M.B., Michowitz Y., Auricchio A., Barbash I.M., Barrabes J.A., Boriani G., Braunschweig F., Brignole M. (2021). 2021 ESC Guidelines on cardiac pacing and cardiac resynchronization therapy. Eur. Heart J..

[49] Zou H., Zhu X., Zhang J., Wang Y., Wu X., Liu F., Xie X., Chen X. (2017). Reference equations for the six-minute walk distance in the healthy Chinese population aged 18-59 years. PLoS ONE.

[50] Uszko-Lencer N., Mesquita R., Janssen E., Werter C., Brunner-La Rocca H.P., Pitta F., Wouters E.F.M., Spruit M.A. (2017). Reliability, construct validity and determinants of 6-minute walk test performance in patients with chronic heart failure. Int. J. Cardiol..

[51] Harris K.M., Anderson D.R., Landers J.D., Emery C.F. (2017). Utility of Walk Tests in Evaluating Functional Status Among Participants in an Outpatient Cardiac Rehabilitation Program. J. Cardiopulm. Rehabil. Prev..

[52] Guyatt G.H., Sullivan M.J., Thompson P.J., Fallen E.L., Pugsley S.O., Taylor D.W., Berman L.B. (1985). The 6-minute walk: A new measure of exercise capacity in patients with chronic heart failure. Can. Med. Assoc. J..

[53] Lee R., Chan Y.H., Wong J., Lau D., Ng K. (2007). The 6-minute walk test predicts clinical outcome in Asian patients with chronic congestive heart failure on contemporary medical therapy: A study of the multiracial population in Singapore. Int. J. Cardiol..

[54] Rostagno C., Olivo G., Comeglio M., Boddi V., Banchelli M., Galanti G., Gensini G.F. (2003). Prognostic value of 6-minute walk corridor test in patients with mild to moderate heart failure: Comparison with other methods of functional evaluation. Eur. J. Heart Fail..

[55] Wilkoff B.L., Corey J., Blackburn G. (1989). A Mathematical Model of the Cardiac Chronotropic Response to Exercise. J. Electrophysiol..

[56] Witte K.K., Cleland J.G., Clark A.L. (2006). Chronic heart failure, chronotropic incompetence, and the effects of beta blockade. Heart.

[57] Khan M.N., Pothier C.E., Lauer M.S. (2005). Chronotropic incompetence as a predictor of death among patients with normal electrograms taking beta blockers (metoprolol or atenolol). Am. J. Cardiol..

[58] Brubaker P.H., Kitzman D.W. (2011). Chronotropic incompetence: Causes, consequences, and management. Circulation.

[59] Zweerink A., van der Lingen A.C.J., Handoko M.L., van Rossum A.C., Allaart C.P. (2018). Chronotropic Incompetence in Chronic Heart Failure. Circ. Heart Fail..

[60] Benes J., Kotrc M., Borlaug B.A., Lefflerova K., Jarolim P., Bendlova B., Jabor A., Kautzner J., Melenovsky V. (2013). Resting heart rate and heart rate reserve in advanced heart failure have distinct pathophysiologic correlates and prognostic impact: A prospective pilot study. JACC Heart Fail..

[61] Dobre D., Zannad F., Keteyian S.J., Stevens S.R., Rossignol P., Kitzman D.W., Landzberg J., Howlett J., Kraus W.E., Ellis S.J. (2013). Association between resting heart rate, chronotropic index, and long-term outcomes in patients with heart failure receiving beta-blocker therapy: Data from the HF-ACTION trial. Eur. Heart J..

[62] Agostoni P., Paolillo S., Mapelli M., Gentile P., Salvioni E., Veglia F., Bonomi A., Corra U., Lagioia R., Limongelli G. (2018). Multiparametric prognostic scores in chronic heart failure with reduced ejection fraction: A long-term comparison. Eur. J. Heart Fail..

[63] Levy W.C., Dardas T.F. (2018). Comparison of cardiopulmonary-based risk models with a clinical heart failure risk model. Eur. J. Heart Fail..

[64] Nutter A.L., Tanawuttiwat T., Silver M.A. (2010). Evaluation of 6 prognostic models used to calculate mortality rates in elderly heart failure patients with a fatal heart failure admission. Congest. Heart Fail..

[65] Baughman K.L. (2002). B-type natriuretic peptide -- a window to the heart. N. Engl. J. Med..

[66] Hartmann F., Packer M., Coats A.J., Fowler M.B., Krum H., Mohacsi P., Rouleau J.L., Tendera M., Castaigne A., Anker S.D. (2004). Prognostic impact of plasma N-terminal pro-brain natriuretic peptide in severe chronic congestive heart failure: A substudy of the Carvedilol Prospective Randomized Cumulative Survival (COPERNICUS) trial. Circulation.

[67] Berezin A.E. (2016). Prognostication in Different Heart Failure Phenotypes: The Role of Circulating Biomarkers. J. Circ. Biomark..

[68] Wang J., Li Z., Chen J., Zhao H., Luo L., Chen C., Xu X., Zhang W., Gao K., Li B. (2013). Metabolomic identification of diagnostic plasma biomarkers in humans with chronic heart failure. Mol. Biosyst..

[69] Lekavich C.L., Barksdale D.J., Neelon V., Wu J.R. (2015). Heart failure preserved ejection fraction (HFpEF): An integrated and strategic review. Heart Fail. Rev..

[70] Zile M.R., Baicu C.F. (2013). Biomarkers of diastolic dysfunction and myocardial fibrosis: Application to heart failure with a preserved ejection fraction. J. Cardiovasc. Transl. Res..

[71] D’Elia E., Vaduganathan M., Gori M., Gavazzi A., Butler J., Senni M. (2015). Role of biomarkers in cardiac structure phenotyping in heart failure with preserved ejection fraction: Critical appraisal and practical use. Eur. J. Heart Fail..

[72] Wilcox J.E., Fonarow G.C., Ardehali H., Bonow R.O., Butler J., Sauer A.J., Epstein S.E., Khan S.S., Kim R.J., Sabbah H.N. (2015). “Targeting the Heart” in Heart Failure: Myocardial Recovery in Heart Failure with Reduced Ejection Fraction. JACC Heart Fail..

[73] Hillege H.L., Nitsch D., Pfeffer M.A., Swedberg K., McMurray J.J., Yusuf S., Granger C.B., Michelson E.L., Ostergren J., Cornel J.H. (2006). Renal function as a predictor of outcome in a broad spectrum of patients with heart failure. Circulation.

[74] Guazzi M., Wilhelm M., Halle M., Van Craenenbroeck E., Kemps H., de Boer R.A., Coats A.J.S., Lund L., Mancini D., Borlaug B. (2022). Exercise testing in heart failure with preserved ejection fraction: An appraisal through diagnosis, pathophysiology and therapy—A clinical consensus statement of the Heart Failure Association and European Association of Preventive Cardiology of the European Society of Cardiology. Eur. J. Heart Fail..

[75] Guazzi M., Bandera F., Ozemek C., Systrom D., Arena R. (2017). Cardiopulmonary Exercise Testing: What Is its Value?. J. Am. Coll. Cardiol..

[76] Guazzi M., Arena R., Halle M., Piepoli M.F., Myers J., Lavie C.J. (2018). 2016 focused update: Clinical recommendations for cardiopulmonary exercise testing data assessment in specific patient populations. Eur. Heart J..

[77] Piepoli M.F., Corra U., Agostoni P. (2017). Cardiopulmonary Exercise Testing in Patients with Heart Failure with Specific Comorbidities. Ann. Am. Thorac. Soc..

[78] Skalski J., Allison T.G., Miller T.D. (2012). The safety of cardiopulmonary exercise testing in a population with high-risk cardiovascular diseases. Circulation.

[79] Ross R., Blair S.N., Arena R., Church T.S., Despres J.P., Franklin B.A., Haskell W.L., Kaminsky L.A., Levine B.D., Lavie C.J. (2016). Importance of Assessing Cardiorespiratory Fitness in Clinical Practice: A Case for Fitness as a Clinical Vital Sign: A Scientific Statement from the American Heart Association. Circulation.

[80] Gibbons R.J., Balady G.J., Beasley J.W., Bricker J.T., Duvernoy W.F., Froelicher V.F., Mark D.B., Marwick T.H., McCallister B.D., Thompson P.D. (1997). ACC/AHA guidelines for exercise testing: Executive summary. A report of the American College of Cardiology/American Heart Association Task Force on Practice Guidelines (Committee on Exercise Testing). Circulation.

[81] Fletcher G.F., Ades P.A., Kligfield P., Arena R., Balady G.J., Bittner V.A., Coke L.A., Fleg J.L., Forman D.E., Gerber T.C. (2013). Exercise standards for testing and training: A scientific statement from the American Heart Association. Circulation.

[82] Roger V.L., Jacobsen S.J., Pellikka P.A., Miller T.D., Bailey K.R., Gersh B.J. (1998). Prognostic value of treadmill exercise testing: A population-based study in Olmsted County, Minnesota. Circulation.

[83] Balady G.J., Arena R., Sietsema K., Myers J., Coke L., Fletcher G.F., Forman D., Franklin B., Guazzi M., Gulati M. (2010). Clinician’s Guide to cardiopulmonary exercise testing in adults: A scientific statement from the American Heart Association. Circulation.

[84] Corra U., Agostoni P.G., Anker S.D., Coats A.J.S., Crespo Leiro M.G., de Boer R.A., Harjola V.P., Hill L., Lainscak M., Lund L.H. (2018). Role of cardiopulmonary exercise testing in clinical stratification in heart failure. A position paper from the Committee on Exercise Physiology and Training of the Heart Failure Association of the European Society of Cardiology. Eur. J. Heart Fail..

[85] Leclerc K. (2017). Cardiopulmonary exercise testing: A contemporary and versatile clinical tool. Cleve Clin. J. Med..

[86] American Thoracic S., American College of Chest P. (2003). ATS/ACCP Statement on cardiopulmonary exercise testing. Am. J. Respir. Crit. Care Med..

[87] Finet J.E., Van Iterson E.H., Wilson Tang W.H. (2021). Invasive Hemodynamic and Metabolic Evaluation of HFpEF. Curr. Treat. Options Cardiovasc. Med..

[88] Glaab T., Taube C. (2022). Practical guide to cardiopulmonary exercise testing in adults. Respir. Res..

[89] Triantafyllidi H., Birmpa D., Benas D., Trivilou P., Fambri A., Iliodromitis E.K. (2022). Cardiopulmonary Exercise Testing: The ABC for the Clinical Cardiologist. Cardiology.

[90] Agostoni P., Dumitrescu D. (2019). How to perform and report a cardiopulmonary exercise test in patients with chronic heart failure. Int. J. Cardiol..

[91] Dumitrescu D., Rosenkranz S. (2017). Graphical Data Display for Clinical Cardiopulmonary Exercise Testing. Ann. Am. Thorac. Soc..

[92] Aaronson K.D., Mancini D.M. (1995). Is percentage of predicted maximal exercise oxygen consumption a better predictor of survival than peak exercise oxygen consumption for patients with severe heart failure?. J. Heart Lung Transplant..

[93] Cohen-Solal A., Tabet J.Y., Logeart D., Bourgoin P., Tokmakova M., Dahan M. (2002). A non-invasively determined surrogate of cardiac power (‘circulatory power’) at peak exercise is a powerful prognostic factor in chronic heart failure. Eur. Heart J..

[94] Agostoni P., Corra U., Cattadori G., Veglia F., Battaia E., La Gioia R., Scardovi A.B., Emdin M., Metra M., Sinagra G. (2013). Prognostic value of indeterminable anaerobic threshold in heart failure. Circ. Heart Fail..

[95] Chua T.P., Ponikowski P., Harrington D., Anker S.D., Webb-Peploe K., Clark A.L., Poole-Wilson P.A., Coats A.J. (1997). Clinical correlates and prognostic significance of the ventilatory response to exercise in chronic heart failure. J. Am. Coll. Cardiol..

[96] Kleber F.X., Vietzke G., Wernecke K.D., Bauer U., Opitz C., Wensel R., Sperfeld A., Glaser S. (2000). Impairment of ventilatory efficiency in heart failure: Prognostic impact. Circulation.

[97] Arena R., Myers J., Abella J., Peberdy M.A., Bensimhon D., Chase P., Guazzi M. (2007). Development of a ventilatory classification system in patients with heart failure. Circulation.

[98] Arena R., Myers J., Aslam S.S., Varughese E.B., Peberdy M.A. (2004). Peak VO_2_ and VE/VCO_2_ slope in patients with heart failure: A prognostic comparison. Am. Heart J..

[99] Hollenberg M., Tager I.B. (2000). Oxygen uptake efficiency slope: An index of exercise performance and cardiopulmonary reserve requiring only submaximal exercise. J. Am. Coll. Cardiol..

[100] Baba R., Nagashima M., Goto M., Nagano Y., Yokota M., Tauchi N., Nishibata K. (1996). Oxygen uptake efficiency slope: A new index of cardiorespiratory functional reserve derived from the relation between oxygen uptake and minute ventilation during incremental exercise. J. Am. Coll. Cardiol..

[101] Davies L.C., Wensel R., Georgiadou P., Cicoira M., Coats A.J., Piepoli M.F., Francis D.P. (2006). Enhanced prognostic value from cardiopulmonary exercise testing in chronic heart failure by non-linear analysis: Oxygen uptake efficiency slope. Eur. Heart J..

[102] Matsumoto A., Itoh H., Eto Y., Kobayashi T., Kato M., Omata M., Watanabe H., Kato K., Momomura S. (2000). End-tidal CO_2_ pressure decreases during exercise in cardiac patients: Association with severity of heart failure and cardiac output reserve. J. Am. Coll. Cardiol..

[103] Olson L.J., Arruda-Olson A.M., Somers V.K., Scott C.G., Johnson B.D. (2008). Exercise oscillatory ventilation: Instability of breathing control associated with advanced heart failure. Chest.

[104] Corra U., Pistono M., Mezzani A., Braghiroli A., Giordano A., Lanfranchi P., Bosimini E., Gnemmi M., Giannuzzi P. (2006). Sleep and exertional periodic breathing in chronic heart failure: Prognostic importance and interdependence. Circulation.

[105] Wasserman K., Hansen J.E., Sue D.Y., Whipp B.J., Froelicher V.F. (1987). Principles of Exercise Testing and Interpretation. J. Cardiopulm. Rehabil. Prev..

[106] Takken T., Mylius C.F., Paap D., Broeders W., Hulzebos H.J., Van Brussel M., Bongers B.C. (2019). Reference values for cardiopulmonary exercise testing in healthy subjects—An updated systematic review. Expert Rev. Cardiovasc. Ther..

[107] Myers J., Kaminsky L.A., Lima R., Christle J.W., Ashley E., Arena R. (2017). A Reference Equation for Normal Standards for VO(2) Max: Analysis from the Fitness Registry and the Importance of Exercise National Database (FRIEND Registry). Prog. Cardiovasc. Dis..

[108] Hansen J.E., Sue D.Y., Wasserman K. (1984). Predicted values for clinical exercise testing. Am. Rev. Respir. Dis..

[109] Dorken Gallastegi A., Ergi G.D., Kahraman U., Yagmur B., Cinar E., Karapolat H., Nalbantgil S., Engin C., Yagdi T., Ozbaran M. (2022). Prognostic Value of Cardiopulmonary Exercise Test Parameters in Ventricular Assist Device Therapy. ASAIO J..

[110] Kaminsky L.A., Arena R., Myers J., Peterman J.E., Bonikowske A.R., Harber M.P., Medina Inojosa J.R., Lavie C.J., Squires R.W. (2022). Updated Reference Standards for Cardiorespiratory Fitness Measured with Cardiopulmonary Exercise Testing: Data from the Fitness Registry and the Importance of Exercise National Database (FRIEND). Mayo Clin. Proc..

[111] Weber K.T., Kinasewitz G.T., Janicki J.S., Fishman A.P. (1982). Oxygen utilization and ventilation during exercise in patients with chronic cardiac failure. Circulation.

[112] O’Neill J.O., Young J.B., Pothier C.E., Lauer M.S. (2005). Peak oxygen consumption as a predictor of death in patients with heart failure receiving beta-blockers. Circulation.

[113] Peterson L.R., Schechtman K.B., Ewald G.A., Geltman E.M., Meyer T., Krekeler P., Rogers J.G. (2003). The effect of beta-adrenergic blockers on the prognostic value of peak exercise oxygen uptake in patients with heart failure. J. Heart Lung Transplant..

[114] Cohn J.N., Rector T.S. (1988). Prognosis of congestive heart failure and predictors of mortality. Am. J. Cardiol..

[115] Keteyian S.J., Patel M., Kraus W.E., Brawner C.A., McConnell T.R., Pina I.L., Leifer E.S., Fleg J.L., Blackburn G., Fonarow G.C. (2016). Variables Measured During Cardiopulmonary Exercise Testing as Predictors of Mortality in Chronic Systolic Heart Failure. J. Am. Coll. Cardiol..

[116] Dhakal B.P., Malhotra R., Murphy R.M., Pappagianopoulos P.P., Baggish A.L., Weiner R.B., Houstis N.E., Eisman A.S., Hough S.S., Lewis G.D. (2015). Mechanisms of exercise intolerance in heart failure with preserved ejection fraction: The role of abnormal peripheral oxygen extraction. Circ. Heart Fail..

[117] Haykowsky M.J., Brubaker P.H., John J.M., Stewart K.P., Morgan T.M., Kitzman D.W. (2011). Determinants of exercise intolerance in elderly heart failure patients with preserved ejection fraction. J. Am. Coll. Cardiol..

[118] Ciani O., Piepoli M., Smart N., Uddin J., Walker S., Warren F.C., Zwisler A.D., Davos C.H., Taylor R.S. (2018). Validation of Exercise Capacity as a Surrogate Endpoint in Exercise-Based Rehabilitation for Heart Failure: A Meta-Analysis of Randomized Controlled Trials. JACC Heart Fail..

[119] De Marco T., Wolfel E., Feldman A.M., Lowes B., Higginbotham M.B., Ghali J.K., Wagoner L., Kirlin P.C., Kennett J.D., Goel S. (2008). Impact of cardiac resynchronization therapy on exercise performance, functional capacity, and quality of life in systolic heart failure with QRS prolongation: COMPANION trial sub-study. J. Card. Fail..

[120] Gitt A.K., Wasserman K., Kilkowski C., Kleemann T., Kilkowski A., Bangert M., Schneider S., Schwarz A., Senges J. (2002). Exercise anaerobic threshold and ventilatory efficiency identify heart failure patients for high risk of early death. Circulation.

[121] Chaudhry S., Arena R., Wasserman K., Hansen J.E., Lewis G.D., Myers J., Chronos N., Boden W.E. (2009). Exercise-induced myocardial ischemia detected by cardiopulmonary exercise testing. Am. J. Cardiol..

[122] Shafiq A., Brawner C.A., Aldred H.A., Lewis B., Williams C.T., Tita C., Schairer J.R., Ehrman J.K., Velez M., Selektor Y. (2016). Prognostic value of cardiopulmonary exercise testing in heart failure with preserved ejection fraction. The Henry Ford HospITal CardioPulmonary EXercise Testing (FIT-CPX) project. Am. Heart J..

[123] Franciosa J.A., Leddy C.L., Wilen M., Schwartz D.E. (1984). Relation between hemodynamic and ventilatory responses in determining exercise capacity in severe congestive heart failure. Am. J. Cardiol..

[124] Malhotra R., Bakken K., D’Elia E., Lewis G.D. (2016). Cardiopulmonary Exercise Testing in Heart Failure. JACC Heart Fail..

[125] Johnson R.L. (2001). Gas exchange efficiency in congestive heart failure II. Circulation.

[126] Lewis G.D., Shah R.V., Pappagianopolas P.P., Systrom D.M., Semigran M.J. (2008). Determinants of ventilatory efficiency in heart failure: The role of right ventricular performance and pulmonary vascular tone. Circ. Heart Fail..

[127] Jaussaud J., Aimable L., Douard H. (2011). The time for a new strong functional parameter in heart failure: The VE/VCO_2_ slope. Int. J. Cardiol..

[128] Cornelis J., Taeymans J., Hens W., Beckers P., Vrints C., Vissers D. (2015). Prognostic respiratory parameters in heart failure patients with and without exercise oscillatory ventilation—A systematic review and descriptive meta-analysis. Int. J. Cardiol..

[129] Arena R., Guazzi M., Cahalin L.P., Myers J. (2014). Revisiting cardiopulmonary exercise testing applications in heart failure: Aligning evidence with clinical practice. Exerc. Sport Sci. Rev..

[130] Nayor M., Xanthakis V., Tanguay M., Blodgett J.B., Shah R.V., Schoenike M., Sbarbaro J., Farrell R., Malhotra R., Houstis N.E. (2020). Clinical and Hemodynamic Associations and Prognostic Implications of Ventilatory Efficiency in Patients with Preserved Left Ventricular Systolic Function. Circ. Heart Fail..

[131] Forman D.E., Guazzi M., Myers J., Chase P., Bensimhon D., Cahalin L.P., Peberdy M.A., Ashley E., West E., Daniels K.M. (2012). Ventilatory power: A novel index that enhances prognostic assessment of patients with heart failure. Circ. Heart Fail..

[132] Arena R., Myers J., Aslam S.S., Varughese E.B., Peberdy M.A. (2004). Influence of subject effort on the prognostic value of peak VO2 and the VE/VCO2 slope in patients with heart failure. J. Cardiopulm. Rehabil..

[133] Lala A., Shah K.B., Lanfear D.E., Thibodeau J.T., Palardy M., Ambardekar A.V., McNamara D.M., Taddei-Peters W.C., Baldwin J.T., Jeffries N. (2021). Predictive Value of Cardiopulmonary Exercise Testing Parameters in Ambulatory Advanced Heart Failure. JACC Heart Fail..

[134] Dhakal B.P., Murphy R.M., Lewis G.D. (2012). Exercise oscillatory ventilation in heart failure. Trends Cardiovasc. Med..

[135] Sun X.G., Hansen J.E., Beshai J.F., Wasserman K. (2010). Oscillatory breathing and exercise gas exchange abnormalities prognosticate early mortality and morbidity in heart failure. J. Am. Coll. Cardiol..

[136] Murphy R.M., Shah R.V., Malhotra R., Pappagianopoulos P.P., Hough S.S., Systrom D.M., Semigran M.J., Lewis G.D. (2011). Exercise oscillatory ventilation in systolic heart failure: An indicator of impaired hemodynamic response to exercise. Circulation.

[137] Stringer W.W., Hansen J.E., Wasserman K. (1997). Cardiac output estimated noninvasively from oxygen uptake during exercise. J. Appl. Physiol. (1985).

[138] Hansen J.E., Sue D.Y., Oren A., Wasserman K. (1987). Relation of oxygen uptake to work rate in normal men and men with circulatory disorders. Am. J. Cardiol..

[139] Vainshelboim B., Kokkinos P., Myers J. (2019). Prognostic Value and Clinical Usefulness of the Hemodynamic Gain Index in Men. Am. J. Cardiol..

[140] Vainshelboim B., Kokkinos P., Myers J. (2020). Hemodynamic gain index in women: A validation study. Int. J. Cardiol..

[141] Chaikijurajai T., Wu Y., Grodin J.L., Harb S., Jaber W., Tang W.H.W. (2022). Validation of prognostic value of the hemodynamic gain index in different groups of patients undergoing exercise stress testing. Am. Heart J. Plus..

[142] Gobel F.L., Norstrom L.A., Nelson R.R., Jorgensen C.R., Wang Y. (1978). The rate-pressure product as an index of myocardial oxygen consumption during exercise in patients with angina pectoris. Circulation.

[143] Mehra M.R., Canter C.E., Hannan M.M., Semigran M.J., Uber P.A., Baran D.A., Danziger-Isakov L., Kirklin J.K., Kirk R., Kushwaha S.S. (2016). The 2016 International Society for Heart Lung Transplantation listing criteria for heart transplantation: A 10-year update. J. Heart Lung Transplant..

[144] Agostoni P., Corra U., Cattadori G., Veglia F., La Gioia R., Scardovi A.B., Emdin M., Metra M., Sinagra G., Limongelli G. (2013). Metabolic exercise test data combined with cardiac and kidney indexes, the MECKI score: A multiparametric approach to heart failure prognosis. Int. J. Cardiol..

[145] Guazzi M., Boracchi P., Arena R., Myers J., Vicenzi M., Peberdy M.A., Bensimhon D., Chase P., Reina G. (2010). Development of a cardiopulmonary exercise prognostic score for optimizing risk stratification in heart failure: The (P)e(R)i(O)dic (B)reathing during (E)xercise (PROBE) study. J. Card. Fail..

[146] Yardley M., Gullestad L., Bendz B., Bjorkelund E., Rolid K., Arora S., Nytroen K. (2017). Long-term effects of high-intensity interval training in heart transplant recipients: A 5-year follow-up study of a randomized controlled trial. Clin. Transplant..

[147] Pelliccia A., Sharma S., Gati S., Back M., Borjesson M., Caselli S., Collet J.P., Corrado D., Drezner J.A., Halle M. (2021). 2020 ESC Guidelines on sports cardiology and exercise in patients with cardiovascular disease. Eur. Heart J..

[148] Myers J., Geiran O., Simonsen S., Ghuyoumi A., Gullestad L. (2003). Clinical and exercise test determinants of survival after cardiac transplantation. Chest.

[149] Yardley M., Havik O.E., Grov I., Relbo A., Gullestad L., Nytroen K. (2016). Peak oxygen uptake and self-reported physical health are strong predictors of long-term survival after heart transplantation. Clin. Transplant..

[150] Tsai H.Y., Tsai W.J., Kuo L.Y., Lin Y.S., Chen B.Y., Lin W.H., Shen S.L., Huang H.Y. (2018). Oxygen Consumption at Anaerobic Threshold Predicts Cardiac Events After Heart Transplantation. Transplant. Proc..

[151] Iglesias D., Masson W., Barbagelata L., Rossi E., Mora M., Cornejo G., Lagoria J., Belziti C., Vulcano N., Marenchino R. (2021). Prognostic value of cardiopulmonary exercise test after heart transplantation. Clin. Transplant..

[152] Grinstein J., Sawalha Y., Medvedofsky D.A., Ahmad S., Hofmeyer M., Rodrigo M., Kadakkal A., Barnett C., Kalantari S., Talati I. (2021). VE/VCO2 slope predicts RV dysfunction and mortality after left ventricular assist device: A fresh look at cardiopulmonary stress testing for prognostication. J. Artif. Organs.

[153] Trombara F., Apostolo A., Vignati C., Naliato M., Ceriani R., Agostoni P. (2020). Effects of left ventricular assist device on cardiopulmonary exercise performance. Eur. J. Heart Fail..

[154] Mancini D., Goldsmith R., Levin H., Beniaminovitz A., Rose E., Catanese K., Flannery M., Oz M. (1998). Comparison of exercise performance in patients with chronic severe heart failure versus left ventricular assist devices. Circulation.

[155] Leibner E.S., Cysyk J., Eleuteri K., El-Banayosy A., Boehmer J.P., Pae W.E. (2013). Changes in the functional status measures of heart failure patients with mechanical assist devices. ASAIO J..

[156] Mirza K.K., Szymanski M.K., Schmidt T., de Jonge N., Brahmbhatt D.H., Billia F., Hsu S., MacGowan G.A., Jakovljevic D.G., Agostoni P. (2021). Prognostic Value of Peak Oxygen Uptake in Patients Supported with Left Ventricular Assist Devices (PRO-VAD). JACC Heart Fail..

[157] Moss N., Rakita V., Lala A., Parikh A., Roldan J., Mitter S.S., Anyanwu A., Campoli M., Burkhoff D., Mancini D.M. (2020). Hemodynamic Response to Exercise in Patients Supported by Continuous Flow Left Ventricular Assist Devices. JACC Heart Fail..

[158] Dunlay S.M., Allison T.G., Pereira N.L. (2014). Changes in cardiopulmonary exercise testing parameters following continuous flow left ventricular assist device implantation and heart transplantation. J. Card. Fail..

[159] Dorken Gallastegi A., Ozturk P., Demir E., Engin C., Nalbantgil S., Yagdi T., Ozbaran M. (2020). Prospective evaluation of ventricular assist device risk scores’ capacity to predict cardiopulmonary exercise parameters. Interact. Cardiovasc. Thorac. Surg..

[160] Rosenbaum A.N., Dunlay S.M., Pereira N.L., Allison T.G., Maltais S., Stulak J.M., Joyce L.D., Kushwaha S.S. (2018). Determinants of Improvement in Cardiopulmonary Exercise Testing After Left Ventricular Assist Device Implantation. ASAIO J..

[161] Maybaum S., Mancini D., Xydas S., Starling R.C., Aaronson K., Pagani F.D., Miller L.W., Margulies K., McRee S., Frazier O.H. (2007). Cardiac improvement during mechanical circulatory support: A prospective multicenter study of the LVAD Working Group. Circulation.

[162] Noor M.R., Bowles C., Banner N.R. (2012). Relationship between pump speed and exercise capacity during HeartMate II left ventricular assist device support: Influence of residual left ventricular function. Eur. J. Heart Fail..

[163] Jung M.H., Hansen P.B., Sander K., Olsen P.S., Rossing K., Boesgaard S., Russell S.D., Gustafsson F. (2014). Effect of increasing pump speed during exercise on peak oxygen uptake in heart failure patients supported with a continuous-flow left ventricular assist device. A double-blind randomized study. Eur. J. Heart Fail..

[164] Mancini D., Gibson G.T. (2021). Survival of the Fittest. JACC Heart Fail..

[165] Tolle J.J., Waxman A.B., Van Horn T.L., Pappagianopoulos P.P., Systrom D.M. (2008). Exercise-induced pulmonary arterial hypertension. Circulation.

[166] Guazzi M., Myers J., Peberdy M.A., Bensimhon D., Chase P., Arena R. (2010). Cardiopulmonary exercise testing variables reflect the degree of diastolic dysfunction in patients with heart failure-normal ejection fraction. J. Cardiopulm. Rehabil. Prev..

[167] Fu Q., Vangundy T.B., Galbreath M.M., Shibata S., Jain M., Hastings J.L., Bhella P.S., Levine B.D. (2010). Cardiac origins of the postural orthostatic tachycardia syndrome. J. Am. Coll. Cardiol..

[168] Lewis G.D., Murphy R.M., Shah R.V., Pappagianopoulos P.P., Malhotra R., Bloch K.D., Systrom D.M., Semigran M.J. (2011). Pulmonary vascular response patterns during exercise in left ventricular systolic dysfunction predict exercise capacity and outcomes. Circ. Heart Fail..

[169] Maron B.A., Cockrill B.A., Waxman A.B., Systrom D.M. (2013). The invasive cardiopulmonary exercise test. Circulation.

[170] Psotka M.A., Abraham W.T., Fiuzat M., Filippatos G., Lindenfeld J., Ahmad T., Felker G.M., Jacob R., Kitzman D.W., Leifer E.S. (2022). Functional and Symptomatic Clinical Trial Endpoints: The HFC-ARC Scientific Expert Panel. JACC Heart Fail..

